# The Paraventricular Thalamus: A Potential Sensor and Integrator of Emotionally Salient Early-Life Experiences

**DOI:** 10.3389/fnbeh.2021.673162

**Published:** 2021-05-17

**Authors:** Cassandra L. Kooiker, Matthew T. Birnie, Tallie Z. Baram

**Affiliations:** ^1^Department of Anatomy & Neurobiology, University of California, Irvine, Irvine, CA, United States; ^2^Department of Pediatrics, University of California, Irvine, Irvine, CA, United States

**Keywords:** paraventricular thalamus, early life adversity, stress, reward, circuit, depression, anxiety

## Abstract

Early-life experiences influence a broad spectrum of behaviors throughout the lifespan that contribute to resilience or vulnerability to mental health disorders. Yet, how emotionally salient experiences early in life are encoded, stored, and processed and the mechanisms by which they influence future behaviors remain poorly understood. The paraventricular nucleus of the thalamus (PVT) is a key structure in modulating positive and negative experiences and behaviors in adults. However, little is known of the PVT’s role in encoding and integrating emotionally salient experiences that occur during neonatal, infancy, and childhood periods. In this review, we (1) describe the functions and connections of the PVT and its regulation of behavior, (2) introduce novel technical approaches to elucidating the role of the PVT in mediating enduring changes in adult behaviors resulting from early-life experiences, and (3) conclude that PVT neurons of neonatal rodents are engaged by both positive and negative emotionally salient experiences, and their activation may enduringly govern future behavior-modulating PVT activity during emotionally salient contexts.

## Introduction

Positive and negative experiences during sensitive developmental periods influence brain maturation to induce lasting alterations to cognitive and emotional behaviors (Fagiolini and Hensch, 2000; Barkat et al., 2011; Callaghan and Richardson, 2011; Danese and Lewis, 2017; Short and Baram, 2019; Levis et al., 2021). Indeed, it is well established that genetics and early-life experiences interact to influence the development of key brain circuits (Klengel et al., 2013; Kundakovic et al., 2013; Di Segni et al., 2016; McIlwrick et al., 2016). However, the mechanisms by which early-life experiences influence future behavior remain unclear. In this review, we discuss the role of the paraventricular nucleus of the thalamus (PVT), a dorsal midline thalamic nucleus, as a sensor and integrator of salient adult experiences, mediating the influence of early-life experiences on future behavior.

## Brief Overview of PVT Anatomy and Functions

The PVT has emerged as an important node in the limbic/reward system and within circuits that control appetitive/approach and aversive/avoidance behaviors (Do-Monte et al., 2015; Zhu et al., 2018; Choi et al., 2019) and is increasingly recognized as a crucial component of the emotional processing network (Barson et al., 2020). The PVT is commonly subdivided by its actions into anterior and posterior parts, with the anterior (a)PVT important in expression of approach behaviors (Browning et al., 2014; Labouèbe et al., 2016; Do-Monte et al., 2017), and the posterior (p)PVT important for avoidance behaviors and responses to chronic stress (Bhatnagar and Dallman, 1998; Heydendael et al., 2011; Pliota et al., 2018).

**FIGURE 1:**
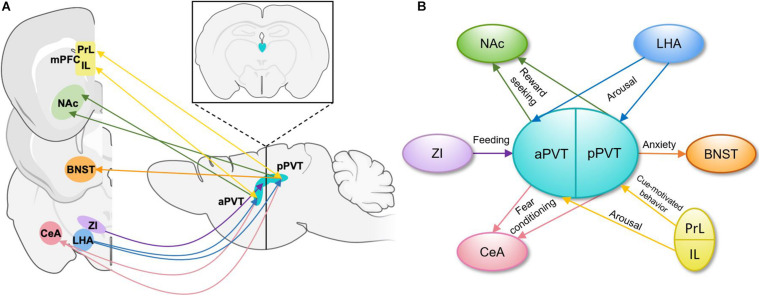
Schematic representations of PVT projections and their functions. **(A)** Input and output patterns of the anterior and posterior PVT, shown in the sagittal plane with an inset image demonstrating the position of the PVT in the coronal plane (Li and Kirouac, 2008; Dong et al., 2017). **(B)** A simplified schematic of functions carried out by specific efferent and afferent aPVT and pPVT projections. aPVT, anterior paraventricular nucleus of the thalamus; BNST, bed nucleus of the stria terminalis; CeA, central nucleus of the amygdala; IL, infralimbic cortex; LHA, lateral hypothalamus; mPFC, medial prefrontal cortex; NAc, nucleus accumbens; pPVT, posterior paraventricular nucleus of the thalamus; PrL, prelimbic cortex; ZI, zona incerta.

The PVT is heterogenous in its afferent and efferent projections and functional output of these projections (Figure 1). PVT projection neurons terminating in the medial shell of the nucleus accumbens (NAc) mediate the retrieval and maintenance of opiate associated memories (Zhu et al., 2016), and inhibition of this projection during retrieval protects against opiate relapse (Keyes et al., 2020). Inhibition of the same projection also decreases stress-induced social avoidance, identifying a complex role for this projection in regulating responses to stress (Dong et al., 2020). The PVT projects strongly to the central amygdala (CeA), and inhibition of this projection decreases sucrose-seeking when an expected sucrose reward is omitted (Do-Monte et al., 2017). Inhibition of this projection also impairs fear-retrieval upon cue presentation in the context of foot shock-induced freezing behavior (Do-Monte et al., 2015) and decreases drug-paired conditioned place preference (Keyes et al., 2020). It is also through this projection that the PVT dampens the activity of the CeA during acute stress (Spencer et al., 2004). In accord with the heterogeneity of the PVT, aPVT projections to the dorsomedial NAc shell and amygdala regulate sucrose-seeking (Labouèbe et al., 2016; Do-Monte et al., 2017; Dong et al., 2017), whereas pPVT projections to the ventromedial NAc shell, bed nucleus of the stria terminalis (BNST), and amygdala modulate cue and context-dependent reward-seeking as well as anxiety-like and fear conditioning behaviors (Do-Monte et al., 2015; Dong et al., 2017; Keyes et al., 2020).

The PVT receives input from a wide variety of cortical and subcortical areas involved in reward and stress-related behaviors (Figure 1). Glutamatergic prelimbic cortex projections to the PVT modulate motivated behaviors through formation and maintenance of associations between cues and aversive or appetitive stimuli (Do-Monte et al., 2015; Campus et al., 2019; Otis et al., 2019). GABAergic zona incerta (ZI) projection neurons to the PVT increase food intake (Zhang and van den Pol, 2017), whereas orexin/hypocretin expressing projections from the lateral hypothalamus (LHA) increase arousal to influence reward-seeking behaviors (Ren et al., 2018). In addition to these areas related to reward-seeking and fear expression, the PVT has been reported to receive moderate CRH input from the paraventricular nucleus of the hypothalamus (PVN; Hsu and Price, 2009), consistent with the PVT’s established role in responses to stress, though this report does not include information about the function of this projection. It is through the integration of these distinct signals by the diverse populations of PVT neurons and through its discrete projections to specific brain regions that the PVT influences motivated behaviors (Millan et al., 2017; Campus et al., 2019).

## The PVT Contributes to Responses to Remote Emotionally Salient Experiences

The PVT contributes to regulating responses to stress. Acute stressors, such as foot shock (Bubser and Deutch, 1999; Gao et al., 2020), immobilization (Otake et al., 2002), forced swim (Zhu et al., 2011), tail suspension (Gao et al., 2020), and air puff (Spencer et al., 2004), as well as chronic stressors, such as the intermittent cold stress paradigm (Bhatnagar and Dallman, 1999), increase neuronal activity in the pPVT (measured by c-Fos expression or calcium imaging). The activation of PVT neurons denotes encoding of such aversive events, because it influences responses to a subsequent stressor. For instance, nacute stressor activates the pPVT in rats that have experienced a prior chronic stressor, whereas the chronic stressor alone does not. Strong support for the specific role of the PVT in encoding stress is provided by experiments demonstrating that lesioning of the pPVT prevents habituation to recurrent stress (Bhatnagar et al., 2002). The PVT may also contribute to sensitization of responses to novel, acute stressors in rats that have been chronically stressed (Bhatnagar and Dallman, 1998; Heydendael et al., 2011). Thus, the PVT influences responses to stress in the context of the occurrence of a prior stress The role of the PVT in encoding memories of remote stress has been further addressed by Do-Monte et al. (2015), who found that PVT inhibition 7 or 28 days (but not 24 h) after conditioning to an auditory tone associated with a foot shock impaired freezing behavior. These findings suggest that the PVT influences responses to stress in a time-dependent manner, modulating responses only to old or remote aversive experiences.

Importantly, the PVT also mediates responses to remote *appetitive* experiences. It is activated by initial exposure to a variety of drugs of abuse (Millan et al., 2017) and again during renewal of extinguished drug-seeking and presentation of cues associated with drug administration (Franklin and Druhan, 2000; Hamlin et al., 2009). Lesioning of the PVT prevents normal context-induced reinstatement of drug-seeking after 7 days of extinction, but not acquisition or extinction of drug-seeking (Hamlin et al., 2009). In a related experiment, Keyes et al. (2020) reported that inhibition of the PVT-NAc pathway prevented drug-primed relapse at 4 or 14 days of extinction in a conditioned place preference assay but did not prevent the acquisition of drug-associated memories. In each of these experiments, the PVT was required for a behavioral response to an appetitive experience (drug exposure) that had occurred remotely before a period of abstinence. These findings suggest that the PVT may encode and contribute to responses to remote, emotionally salient experiences regardless of their valence (i.e., positive or negative).

## Neuromodulators and the PVT

A variety of neurotransmitters, neuromodulators, and their receptors are expressed in the PVT, and they are commonly expressed in a gradient across the structure’s anteroposterior axis. Thus, in mice, the dopamine D1 receptor is more densely expressed in the aPVT while the dopamine D2 receptor (D2R) is more densely expressed in the pPVT (Gao et al., 2020). Approximately two thirds of PVT neurons express D2R, with evidence suggesting that increased D2R signaling in the PVT decreases cocaine sensitization (Clark et al., 2017). Other crucial receptors in the PVT are acted upon by neuropeptide ligands, and these receptors and their ligands are often also expressed in gradients across the PVT. Neurotensin receptor type 1 and 2 are found in the PVT, as well their ligand, a neuropeptide that is expressed throughout the PVT but most densely at its anterior and posterior ends (Boudin et al., 1996; Sarret et al., 2003; Curtis et al., 2021). Neurotensin acts in the pPVT to decrease ethanol consumption and in the aPVT to increase exploratory behavior following chronic alcohol intake (Pandey et al., 2019; Pandey and Barson, 2020). Though its cognate receptor is yet undescribed, the neuropeptide cocaine and amphetamine related transcript (CART), is highly expressed in the mouse aPVT (Curtis et al., 2021). CART attenuates drug-primed reinstatement of drug-seeking when injected into the PVT (James et al., 2010), suggesting a role in inhibition of reward-seeking. Receptors to orexin, a neuropeptide with important roles in reinforcing properties of drugs of abuse (Martin-Fardon and Boutrel, 2012), are found throughout the PVT, and blockade of these receptors in rats decreases anxiety-like behavior in the elevated plus-maze (Heydendael et al., 2011) but does not affect expression of conditioned fear (Dong et al., 2015). In addition, microinjection of orexin into the pPVT increases avoidance and anxiety-like behaviors (Li et al., 2010a, b) whereas microinjection into the aPVT increases consumption of ethanol, but not sucrose (Barson et al., 2015), indicating that the role of orexin in the PVT may vary based on anatomical location.

Corticotropin-releasing hormone receptor type 1 (CRHR1) and type 2 (CRHR2), which regulate behavioral, autonomic, endocrine, and immune responses to stress, are also expressed in the PVT (Eghbal-Ahmadi et al., 1998; Chen et al., 2000). Their ligand, the neuropeptide CRH, is present throughout the PVT, with slightly higher density in the pPVT (Itoga et al., 2019). In addition to its established role as a hypothalamic neurohormone, CRH is a key stress-reactive neuropeptide that is expressed in nodes involved in reward and stress, such as the BNST, hippocampus, amygdala, VTA, and NAc (Joëls and Baram, 2009; Grieder et al., 2014; Deussing and Chen, 2018). Notably, all of these structures receive projections from the PVT (Dong et al., 2017). Recently, photoactivation of CRH + aPVT projection neurons targeting the NAc was shown to increase avoidance in the predator-odor task and to reduce reward-seeking, suggesting that CRH + PVT neurons regulate approach and avoidance behaviors (Do-Monte et al., 2020).

Each of these neuromodulators or receptors contribute to particular functional or behavioral niches occupied by the PVT, including those related to drug-seeking, approach and avoidance behaviors, and responses to stress. Consequently, they likely contribute to the PVT’s roles in responses to remote emotionally salient experiences as well.

## Diverse Consequences of Early-Life Experiences on the Brain and Behavior

Both positive and negative early life experiences exert enduring consequences on motivated behaviors as well as on responses to stress later in life (Hyman, 2009; Chen and Baram, 2016; Novick et al., 2018; Raymond et al., 2018). More specifically, early life adversity (ELA) due to trauma, poverty, or tumultuous environment is associated with poor cognitive and emotional health and increased risk for affective disorders, such as depression, schizophrenia, and addiction (Heim et al., 2008; Enoch, 2011; Grassi-Oliveira et al., 2016; Birnie et al., 2020). Notably, ELA exerts sexually dimorphic long-term effects. Preclinical studies indicate increased drug-seeking behavior and palatable food consumption in females (Machado et al., 2013; Levis et al., 2019) and a reduction in these behaviors in males (Bolton et al., 2018b; Ordoñes Sanchez et al., 2021). In both sexes, little is known of the contribution of the PVT in influencing reward and stress-related behaviors following early-life emotionally salient experiences, because studies of the PVT’s role in these changes were performed in adults.

Many of the adult behaviors associated with early life experiences involve the functions of the PVT, indicating a possible role for this region in contributing to the observed deficits. For example, anhedonia, the reduced ability to experience pleasure derived from otherwise enjoyable activities, is a predictive sign of depression, schizophrenia, and substance use disorders (Gorwood, 2008). Anhedonia is observed in rodent models of ELA, manifesting as decreased social play and decreased consumption of natural and drug rewards (Molet et al., 2016; Bolton et al., 2018a, b). Manipulation of the PVT recapitulates these deficits of reward-seeking. For example, photoactivation of aPVT projections to the NAc decreases sucrose-seeking (Do-Monte et al., 2017), and tetanus toxin-mediated blockade of PVT-NAc synaptic transmission decreases cocaine self-administration (Neumann et al., 2016). ELA induces addiction-like behaviors in females, including increased opioid relapse-like behavior and increased demand for opioids in the Behavioral Economics task, i.e., persistent lever pressing for opioid administration despite increasing “cost” (the number of presses for a given dose, Levis et al., 2019). ELA also augments cue-induced relapse-like behaviors to cocaine (Lynch et al., 2005). Manipulations of PVT function recapitulate such addiction-like behaviors. pPVT inhibition prevents cocaine cue-induced relapse-like behaviors (Matzeu et al., 2015), and inhibition of anterior and mid-PVT projections to the NAc prevents opioid-primed relapse and blocks preference for a morphine-paired chamber in a morphine-conditioned place preference assay (Keyes et al., 2020). Similar to the influence of ELA on alcohol dependence, which includes increased intermittent access consumption (Daoura et al., 2011), accelerated intake escalation, and exacerbated affective dysfunction during withdrawal (Okhuarobo et al., 2020), stimulation of the aPVT with orexin or substance P increases intermittent-access consumption of alcohol (Barson et al., 2015).

In addition to disruptions in reward circuit function, ELA also induces perturbations in fear memory and expression, including enhanced freezing behavior in both auditory and contextual fear conditioning paradigms (Champagne, 2008; Sampath et al., 2014; Arp et al., 2016). Circuits mediating conditioned fear learning are traditionally not thought to include the PVT. Yet, the PVT may contribute to such behaviors. For example, inhibition of pPVT projections to the CeA during fear conditioning or fear memory retrieval impairs freezing behavior, suggesting that this projection regulates the establishment and expression of fear memory (Penzo et al., 2015). In summary, there is a significant congruence of behavioral outcomes of ELA and the effects of manipulation of PVT activity, and this observation is likely not coincidental.

**FIGURE 2:**
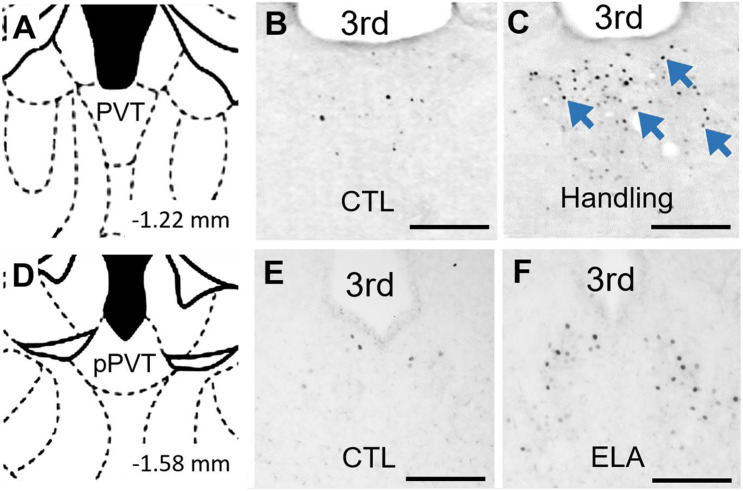
PVT neurons are activated by early life experiences. **(A)** Level of mid PVT indicated by brain atlas (Paxinos and Watson, 2005). **(B)** In P9 rats that were reared in control conditions through P2-9, little activation of PVT was found on P9. **(C)** In contrast, abundant neuronal activation in PVT (blue arrows) was observed 30 min after receiving augmented maternal care in P9 in rats handled from P2-9 (Handling). Adapted from Fenoglio et al. (2006) with permission. **(D)** Level of posterior PVT indicated by brain atlas. **(E)** Little activation of PVT was observed in P9 mice reared in control conditions through P9. **(F)** In contrast, abundant neuronal activation in PVT was observed on the morning of P9 in mice reared in ELA conditions beginning on P2. 3rd, third ventricle. Scale bars, 500 um.

**FIGURE 3:**
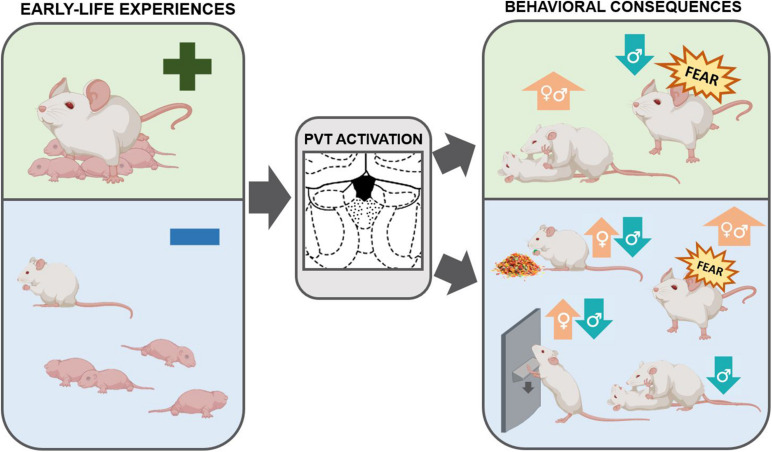
Schematic of the relationship between early life experiences, the PVT, and consequences on adult behaviors. The PVT is engaged by emotionally-salient early-life experiences, both positive and negative. This activation may have enduring consequences in shaping behaviors later in life. Optimal early life experiences result in increased social play in both males and females and decreased fear expression in males. By contrast, ELA, as compared to control rearing conditions or optimal early life experiences, increases palatable food consumption and drug-seeking behaviors in females. ELA decreases social play, palatable food consumption, and drug-seeking in males and enhances fear expression in both sexes. These behavioral consequences may be mediated by activity in the PVT (Caldji et al., 1998; Arp et al., 2016; Molet et al., 2016; Bolton et al., 2018a, b, 2019; Levis et al., 2019; Ordoñes Sanchez et al., 2021).

Indeed, as the PVT plays a crucial role in influencing behavior in response to remote emotionally salient events, might it contribute to the effects of experiences as remote as those early in life? Our group finds that a week of recurrent bouts of augmented maternal care during early postnatal development increased c-Fos expression in the PVT relative to that of pups reared in control conditions (Fenoglio et al., 2006). This activation of the PVT by salient early-life experiences is not limited to positive experiences: Recent observations from our group indicate that c-Fos expression in the PVT is increased by ELA (Figure 2). Thus, PVT engagement by both positive and negative emotionally salient experiences occurs even during early postnatal life. This observation supports the plausibility of a role for the PVT in encoding emotionally salient experiences early in life and in mediating consequences of these experiences later in life (Figure 3).

## Experimental Paradigms and Novel Technologies to Identify the Impact of Early Life Experiences

The development of preclinical models of positive and negative early life experience provides researchers the opportunity to understand complex neural mechanisms using approaches that are not possible in humans. Numerous paradigms have been used to generate stress or adversity during sensitive developmental periods. These models generally result in perturbation of the functions of the reward circuit, leading to deficits in reward-seeking behaviors (Ventura et al., 2012; Molet et al., 2014; Bolton et al., 2018a, b; Levis et al., 2019) and, in some cases, anxiety-like or depressive-like behaviors (Daniels et al., 2004; Wang et al., 2012; Goodwill et al., 2019).

One such experimental model of ELA is the long-established recurrent maternal separation model, in which pups are separated from the dam daily for prolonged periods (Hofer, 1973; Rosenfeld et al., 1992; Huot et al., 2004; Leussis et al., 2012; van Bodegom et al., 2017). More recently, the limited bedding and nesting model (LBN), in which pups are raised for a week in impoverished cages, has gained wide acceptance as a paradigm of simulated poverty (Molet et al., 2014). The LBN cage environment stresses the rodent dams and provokes fragmented, unpredictable maternal care behaviors, with a myriad of enduring consequences on pups’ cognitive and emotional-like behaviors (Rice et al., 2008; Chen and Baram, 2016; Krugers et al., 2017; Walker et al., 2017; Goodwill et al., 2019).

Experimental approaches have also been applied for generating positive early-life experiences. Again, because the key source of environmental signals to neonatal rodents (and humans) is the parent, these paradigms have aimed to manipulate maternal caring behaviors. The neonatal handling procedure involves removing the dam for 15 minutes daily, leading to barrages of maternal care upon her return to the cage (Levine et al., 1967; Fenoglio et al., 2004, 2006; Korosi and Baram, 2009; Korosi et al., 2010; Singh-Taylor et al., 2018). Adult mice and rats exposed to daily handling/augmented maternal care demonstrate attenuated stress responses and increased resilience to depressive-like behavior (Liu et al., 1997; Fenoglio et al., 2006; Korosi et al., 2010).

Importantly, the paradigms described above allow for creating a suite of early-life experiences, setting the stage for dissection of the specific neural circuitries and cell populations involved in the resulting behavioral changes - including the role of the PVT.

Current approaches for uncovering the role of the PVT in encoding early-life experiences and mediating their behavioral consequences involve the use of the targeted recombination of active populations (TRAP) technique. This method allows for permanent access to neuronal ensembles activated during a specific experience, enabling assessment of the role of these neurons in reward and stress-related behaviors later in life (Guenthner et al., 2013; DeNardo et al., 2019). Further, the use of genetic manipulation tools such as designer receptors exclusively activated by designer drugs (DREADDs) and opsins allows researchers to dissect brain region and cell-type specific functional control of behavior. Even newer methods, such as ChRmine, a deep brain optogenetic virus, may enable specific activation of defined neural circuits without intracranial surgery, a major advantage for work involving the fragile skull and brain of neonatal mice (Marshel et al., 2019; Chen et al., 2021). These tools and resources can be harnessed to selectively label and manipulate neuronal populations salient to early life, providing exciting avenues for elucidating the mechanisms by which the PVT contributes to the long-term behavioral outcomes of early-life experiences.

## Conclusion

There is a strong association between early-life experiences and subsequent resilience or vulnerability to emotional disorders. Many of these disorders are characterized by disruptions in behaviors related to reward-seeking, fear expression, and stress responses (Insel et al., 2010; Millan et al., 2012; Whitton et al., 2015). The PVT clearly contributes to the effects of remote emotionally salient experiences on such behaviors, yet the consequences of activation of the PVT by different forms of early-life experiences on adult behaviors remain unclear. Emerging evidence suggests that the PVT is engaged by emotionally salient early-life experiences and therefore is a prime candidate to mediate the altered reward and stress-related behaviors resulting from these diverse experiences. Whereas the PVT responds to both positive and negative early-life experiences, it is possible that the valence of these experiences results in engagement of different populations of PVT neurons, each with unique projection targets and gene expression characteristics. If these patterns of activation predict changes in adult behaviors, the behavioral changes mediated by the PVT under each condition may be divergent. Gaining a deeper understanding of the roles of the PVT in encoding and integrating early-life experiences may have substantial implications for identifying targets for prevention of mental illness.

## Author Contributions

CK wrote an initial draft. All authors conceived the manuscript, refined the manuscript, and approved the submitted version.

## Conflict of Interest

The authors declare that the research was conducted in the absence of any commercial or financial relationships that could be construed as a potential conflict of interest.
